# The Effectiveness of Psychological Treatments in Women with Breast Cancer: A Systematic Review and Meta-Analysis

**DOI:** 10.3390/jcm9010209

**Published:** 2020-01-12

**Authors:** Angela Guarino, Cristina Polini, Giuseppe Forte, Francesca Favieri, Ilaria Boncompagni, Maria Casagrande

**Affiliations:** 1 Department of Psychology, “Sapienza” University of Rome, 00185 Rome, Italy; g.forte@uniroma1.it (G.F.); francesca.favieri@uniroma1.it (F.F.); 2 Department of Dynamic and Clinical Psychology, “Sapienza” University of Rome, 00185 Rome, Italy; cristina.polini@gmail.com (C.P.); ilaria.boncompagni@uniroma1.it (I.B.); maria.casagrande@uniroma1.it (M.C.)

**Keywords:** breast cancer, psychological treatment, cognitive behavioural therapy, psycho-educational therapy, supportive expressive therapy, anxiety, depression, mood, quality of life

## Abstract

Breast cancer is the most prevalent oncological disease among women, and it represents the second oncological cause of death. Many studies have considered the quality of life in people with breast cancer because this condition has high comorbidity with mental distress, anxiety, affective disorders and depression. Psychological interventions can reduce the stressful consequences of both the diagnosis and the medical treatments of breast cancer. However, different methods (e.g., group or individual therapy) and focus (e.g., improving personal skills or increasing emotional well-being) do not help to identify which type of psychological therapy can be more effective in improving quality of life in patients with breast cancer. This study was aimed to systematically review and compare, by means of a meta-analysis, the efficacy of cognitive behavioural, supportive-expressive or psycho-educational treatments in women with breast cancer, focusing on anxiety, depression, mood and quality of life as outcomes. The PRISMA statement was adopted. MEDLINE, PsycINFO, PUBMED and PsycArticles databases were used, and reference lists were examined for additional publications. In the selection of the articles were included studies considering women between 18 and 65 years who were diagnosed with breast cancer at any stage and under any treatment, and who underwent psychological group interventions. At the end of the systematic review, 45 studies met all inclusion criteria and were analysed in the meta-analysis. The overall effect size was medium, especially considering cognitive behavioural therapy and psycho-educational treatments. However, the studies are characterised by high methodological heterogeneity. Despite some limitations, this review and meta-analysis partially confirm the efficiency of cognitive-behavioural and psycho-educational therapies in the improvement of well-being in women with breast cancer.

## 1. Introduction

Breast cancer is the most prevalent oncological disease in women, with a lifetime incidence ratio of one in eight [1,2]. It represents the second oncological cause of death [3,4] and twenty-five per cent of all new cancer diagnoses in women, with a higher incidence in the age range from 55 to 64 years [4,5]. Despite the growing and ageing of the global population, and the increasing number of new breast cancer diagnoses, survival rates have been improved due to advancements in the assessment and treatment [1].

Lifespan consequences and quality of life in women with breast cancer have been the most studied [6,7].

Breast cancer shows high a comorbidity rate, with mental distress [8,9], anxiety and affective disorders [8,10], depression [11,12], and chronic fatigue and decreased social interactions being common responses to breast cancer diagnosis and treatment [13,14,15]. Moreover, women with a primary breast cancer diagnosis remain vulnerable to psychological disorders for many years [16,17,18,19], highlighting the high impact of this medical condition in the quality of life of the patients.

Psychological interventions could reduce the distress consequent to both the diagnosis and medical treatment of breast cancer. However, the studies assessing the effectiveness of psychological therapies are characterised by a heterogeneity of methods (e.g., group or individual therapy) and focuses (e.g., improving personal skills or increasing emotional well-being).

Among the different psychological approaches, cognitive behavioural therapy (CBT) is focused on improving the affective state and coping with the disease [20,21] through the modification of maladaptive cognitive schemas and the diminution of personal distress. CBT adopts different strategies (e.g., relaxation training), and it appears to be effective in reducing levels of anxiety and depression [22,23], restructuring negative automatic cognitive schemas [24], and increasing optimism and positive thought [3,25,26], which help to improve the quality of life of patients with breast cancer [6,7].

Other conventional psychological approaches in the treatment of women with breast cancer are the supportive-expressive and psycho-educational therapies, mainly characterised by group interventions focused on the improvement of personal well-being through the increase of positive affectivity and emotional states [27,28,29]. Supportive-expressive group therapy (SET) is a cognitive emotion-focused therapy aimed at promoting the development of social support and emotional expression and the examination of existential concerns [30]. SET appears to reduce emotional distress related to breast cancer [27,30]. Psycho-educational therapy (PET) is an interdisciplinary approach, including an educational program (e.g., providing adequate knowledge concerning the disease and its related treatments) and a psychological intervention (e.g., providing affective and cognitive skills to elaborate the experience related to the illness) [31]. Several studies have shown the efficacy of PET in improving adaptive coping strategies and quality of life, and in reducing the psychopathological symptomatology (as depression and anxiety). It also allows increasing both the treatment compliance and self-efficacy in women with breast cancer [32,33,34].

Scientific evidence (for a review see: [35,36]) confirmed the effectiveness of all these approaches, showing high positive effects in reducing anxiety and depression and improving quality of life. Moreover, these effects are maintained both in short- and long-term follow-ups [35,37,38].

Although previous reviews [39,40,41,42,43] have analysed this topic and highlighted how psychological interventions can ameliorate the quality of life of women with breast cancer, it is still unclear which intervention is the most effective. Generally, single studies and reviews have focused on a specific or limited number of psychological outcomes. However, acquiring information about several outcomes could be useful in order to both define the effectiveness of a specific intervention on the psychological well-being of patients and to develop more functional treatments. Additionally, there are a limited number of studies comparing different approaches. The aim of this study is therefore to systematically review and compare, through a meta-analysis, the effectiveness of different (cognitive behavioural, supportive-expressive, and psycho-educational) group therapies in women with breast cancer, focusing on different psychological outcomes (anxiety, depression, mood, and quality of life) as an expression of patients’ psychological well-being. It was hypothesised that there would be a general efficacy of all these psychological interventions in patients who underwent these group treatments compared to control groups. Moreover, we analysed the differences in the effectiveness of these types of intervention, considering each psychological outcome.

## 2. Methods

### 2.1. Method

The Preferred Reporting Items for Systematic Reviews and Meta-Analyses (PRISMA) statements [44,45] were adopted. Registration of the protocol was not planned.

### 2.2. Research Strategies

Two independent researchers (I.B, C.P.) conducted four electronic bibliographic database searches (PsychINFO, PubMed, Medline, PsycArticles) of the studies testing psychological interventions in women with breast cancer.

A list of keywords and MeSH terms was generated to identify studies falling within three key search strategies: 1. Terms related to breast cancer: “breast cancer”, “breast neoplasm”; 2. Terms related to psychological intervention: “group psychotherapy”, “supportive psychotherapy” “cognitive behaviour therapy”, “expressive psychotherapy”, “psycho-education *”; 3. Terms related to outcomes: “quality of life”, “anxiety”, “depression”, “distress”, “emotional states”.

Restrictions were made, limiting the research to academic publications with English full-text until February 2019. Additionally, the bibliographical references of retrieved articles, reviews, and meta-analyses were screened to assess whether they contained relevant studies to include in the review.

### 2.3. Eligibility Criteria

The studies were eligible for review if they included women between the ages of 18 and 65 who were diagnosed with breast cancer at any stage and under any treatment, and who were submitted to one psychological intervention. Specifically, the principal inclusion criteria were: (1) studies comparing a group who underwent psychological treatment and a control group with the same pathology but not subjected to the intervention (they received alternative intervention, usual care or waitlist); (2) studies which considered psycho-educational treatment (PET), cognitive behavioural therapy (CBT) or supportive-expressive group therapy (SET) aimed at improving quality of life and mood and to reduce anxiety and depression; (3) studies which adopted randomized controlled design and non-randomized controlled design were both included.

Exclusion criteria were: (1) treatments based on online or telephone interventions, intensive residential interventions, or single intensive session interventions; (2) studies considering individual treatment; (3) studies that compared the effectiveness of group interventions with individual treatment; (4) studies with a single-group pre–post comparison.

No restrictions about the provider of the intervention (e.g., oncologist, psychologist, psychiatrist, social worker, nurse, volunteer, or other) or length of follow-up were inserted. Any standardised screening or diagnostic measure could determine the outcome. Studies presenting composite results, methodological criticisms and those that did not report essential data were excluded.

### 2.4. Study Selection

After the removal of duplicates, the initial assessment of eligibility was based on titles and abstracts. Disagreement in selection was resolved by consulting supervisors (A.G. and M.C.). Two authors (G.F., F.F.) independently examined full texts to confirm the suitability of the studies for following qualitative and quantitative synthesis.

### 2.5. Data Collection

According to the PICOS approach (Population, Intervention, Comparator, Outcomes and Study Design) [44], the main information of each study, such as the author(s) and year of publication, size, and characteristics of the sample (cancer stage, condition during treatment), control and intervention groups conditions, provider type, total intervention time and psychological screening tools, were extracted.

Table 1 reports the data.

### 2.6. Data Synthesis

For the quantitative analyses, pre- and post-intervention means and standard deviations of the determined psychological variables were extracted.

### 2.7. Qualitative Assessment

The methodological quality of studies was assessed using the criteria from the Cochrane Handbook for Systematic Review [46] modified ad hoc according to the aims of this systematic review. The following items were included: use of the randomisation process, including allocation of random sequence (selection bias), selection of sample or baseline imbalances of sample group size (selection bias), use of appropriate tasks for the analysis of the psychological variables considered (detection bias), incomplete outcome data (attrition bias), selective reporting (reporting bias) and other biases.

The quality was categorised as unclear/low/high risk of bias (“+” for low risk of bias, “−” for high risk of bias and “?” for unclear risk of bias).

The overall risk of bias was defined according to the level of risk in the various domains. If a study showed a high risk of bias in at least one domain and showed unclear risk in more domains, it was classified as having a high risk of bias; low risk of bias was chosen if all the domains presented a low risk of bias. If a study showed an unclear risk in at least one domain, the study was considered to have some concern of risk of bias.

### 2.8. Quantitative Analysis

The mean effect size (ES) (Cohen’s d) and the 95% confidence interval (CI) for psychological outcomes were computed. Effect size was calculated by considering mean scores of intervention and control groups.

The interpretation of the ES was small (lower than 0.2), medium (between 0.21 and 0.5), and large (higher than 0.8) [47]. Given that the effect estimates from multiple studies show more variance than the effect drawn from a single population, the random effects model (REML) was adopted to estimate effect size, using the Sidik-Jonkman estimator and the Hartung–Knapp-Sidik-Jonkman (HKSJ) method [48]. Heterogeneity between studies was estimated using the I^2^, and t was considered as low (25%), moderate (50%), and high (75%) [49].

Forest plots were used to display the overall ES and weight in percentage, which indicated the influence of the study. Subgroup analyses were conducted to determine which characteristics of both sample and intervention influenced the ES. This procedure also allowed the identification of effects determined by methodological characteristics of the studies (the type of provider, duration of intervention, control condition and inclusion criteria; see Table 1).

All analyses were carried out with R Studio.

## 3. Results

### 3.1. Study Selection

**Figure 1:**
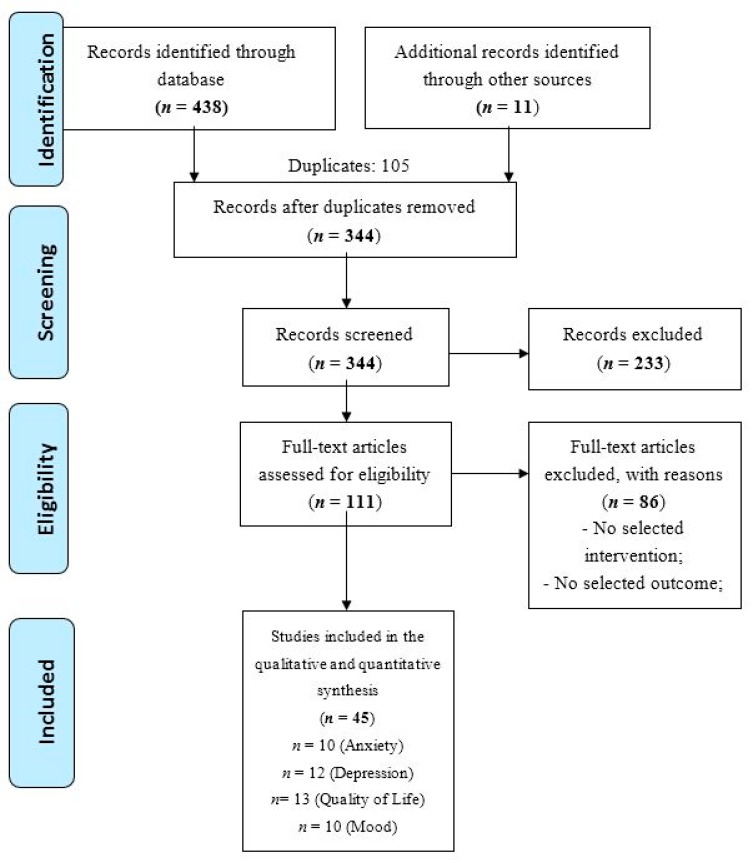
The study screening process.

The flow diagram of the screened studies is presented in Figure 1. Four hundred and forty-nine studies were retrieved from databases (*n* = 438) and other sources (*n* = 11). After excluding duplicates (*n* = 105) and studies not matching inclusion criteria (*n* = 233), 111 full texts were considered for the systematic review. After reading the full-texts, 25 studies from January 2003 to April 2017 met all inclusion criteria and were included in the meta-analysis. Some selected studies analysed the effect of the intervention on different psychological aspects providing distinct outcomes. According to the aims of this study (i.e., to analyse the effectiveness of some psychological interventions in women with breast cancer considering specific psychological aspects such as mood, depression, anxiety and quality of life), studies with multiple outcomes were considered as independent. For these reasons, the meta-analysis included 45 independent studies (see Figure 1 and Table 1).

### 3.2. Characteristics of Included Studies

The characteristics of selected studies are presented in Table 1.

The total number of participants with cancer included in the clinical trials was 8472, considering the pre-intervention evaluation, and 7834 viewing the post-treatment assessment, with a drop-out of around 8% (*n* = 638).

Ten studies considered the effect of treatment on anxiety [24,30,32,50,51,52,53,54,55,56]; twelve studies considered the effect of treatment on depression [24,30,50,51,52,53,54,55,56,57,58,59,60,61]; ten studies considered the effect of the treatment on mood [24,28,32,33,34,57,59,63,66,67]; thirteen studies considered the effects of the treatment on the quality of life [25,32,33,34,51,52,53,54,59,62,63,64,65,66].

Seventeen studies used cognitive behavioural therapy (CBT), four studies used psycho-educational treatment (PET), and four studies analysed supportive-expressive group therapy (SET) (see Table 1 and Table 2).

The providers who implemented the therapies were mainly psychologists, nurses, psychologists and psychiatrists together, as well as mindfulness experts (see Table 1). In some circumstances, the providers were a multidisciplinary team or postgraduate students, while three studies did not give specific information about the providers [58,61,65].

The duration of the interventions ranged from one [65] to twenty weeks [24] (see Table 1).

Several questionnaires were used to measure the psychological variables. To evaluate anxiety, the following questionnaires were used: the State-Trait Anxiety Inventory (STAI) [68], the Hospital Anxiety and Depression Scale (HADS) [69] and the Personality Assessment Inventory (PAI) [70] (see Table 1). For the assessment of depression, the Beck Depression Inventory (BDI) [71], the Centre for Epidemiologic Studies Depression Scale (CES-D) [72], the Functional Assessment of Cancer Therapy (FACT) [73], the PAI [70] and the HADS [69] were employed (see Table 1). Mood was assessed by using the Affect Balance Scale (ABS) [74], the Brief Symptom Inventory (BSI) [75] and the Profile of Mood States (POMS) [76] (see Table 1). For the assessment of quality of life, the following questionnaires were used: the ABS [74], the European Organisation for Research and the Treatment of Cancer Core Quality of Life Questionnaire (EORTC-C30) [77], the FACT [73], the Medical Outcomes Studies Short-Form General Health Survey (MCS-12) [78], the Quality of Life Scales for Korean Patients with Cancer [79], the World Health Organisation-5 Well-Being Index (WHO-5) [80] (see Table 1).

### 3.3. Qualitative Assessment of the Risk of Bias

**Figure 2:**
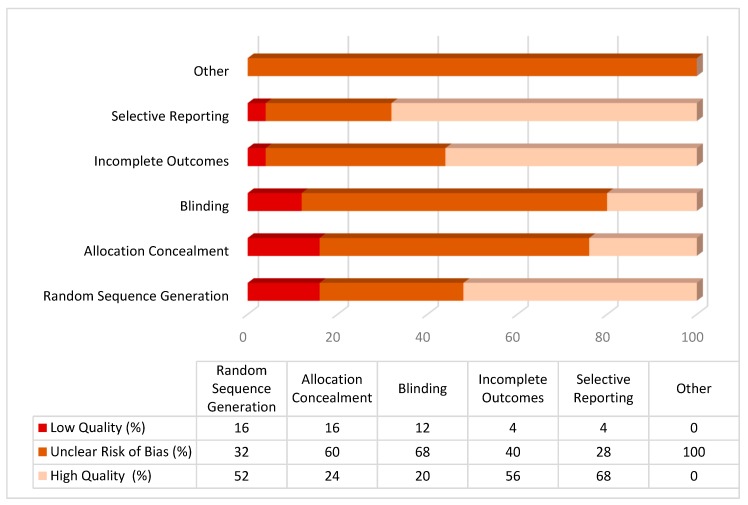
Qualitative assessment (% of studies for each condition of risk of bias).

The risk of bias was assessed. Overall, about one−third of the articles (*n* = 8/25; 32%) showed a high risk of bias on at least one criterion (Table 2). The remaining studies (*n* = 17/25; 68%) presented at least some concerns (e.g., allocation concealment, incomplete outcome, outcome assessment). The highest risks of bias were identified in both the randomisation process and the allocation of participants (*n* = 4/25), while the lowest risk of bias was identified in the selective reports of the data (*n* = 18/28) (Figure 2).

### 3.4. Overall Effect Size Considering Differences between Intervention and Control Group after the Treatment

Overall effect sizes were reported considering both different psychological variables and interventions.

### 3.5. Anxiety

**Figure 3:**
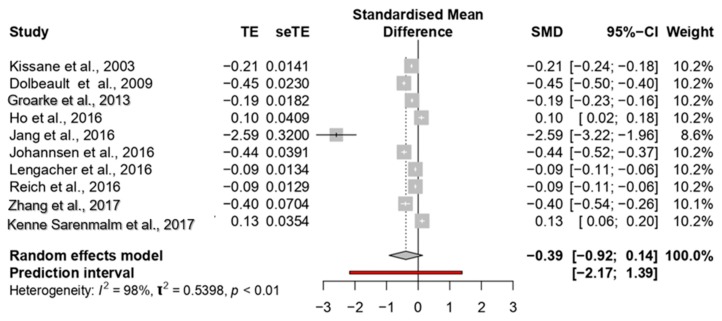
Forest plot of the studies considering psychological interventions focused on reducing anxiety.

The overall ES of the studies about anxiety reduction on women with breast cancer was medium (0.39; 95% CI; −0.91 to 0.14) with a high heterogeneity among the studies (*p* < 0.0001; I^2^ = 98%) (see Figure 3).

The ES ranged between 0.10 [30] and −2.59 [51].

The study of Jang et al. [51] showed the most significant effect size (−2.59); four studies reported medium ESs, ranging between −0.40 [55] and −0.45 [32]. The other studies reported small ESs, ranging between −0.09 [54] and −0.21 [24].

Considering subgroups analyses, significant differences among studies were observed about types of intervention (*p* = 0.001) and types of the provider (*p* = 0.0001). Specifically, studies on CBT and PET interventions showed larger ESs (see Table 3), and larger ESs were observed on studies that included different types of providers (ES = −0.94). No differences were shown considering inclusion criteria of intervention group (*p* = 0.38), duration of intervention (*p* = 0.52) or control group condition (*p* = 0.10).

### 3.6. Depression

**Figure 4:**
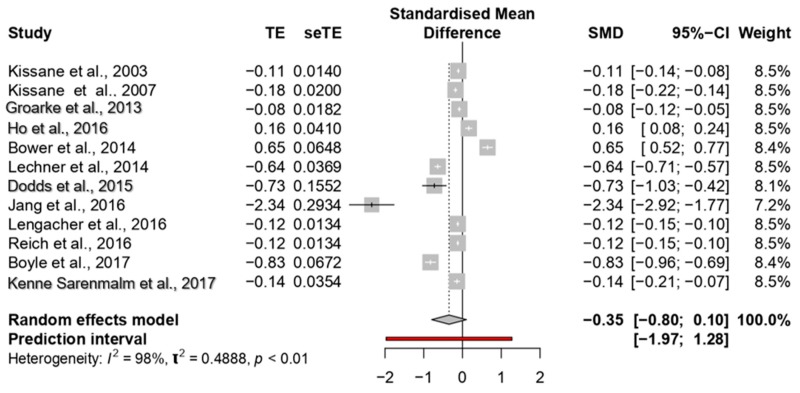
Forest plot of the studies considering psychological interventions focused on reducing depression.

The overall ES of psychological interventions on depression was medium (−0.35; 95% CI: −0.79 to −0.10) (Figure 4). The meta-analysis showed higher heterogeneity among the studies (*p* < 0.0001; I^2^ = 98%) (see Figure 4).

Two studies showed a large ES, ranging between −0.83 [61] and −2.34 [51]; four studies reported medium ESs, ranging between −0.64 [59] and −0.73 [60]. The other studies reported small ESs ranging between −0.08 [50] and −0.18 [57].

Regarding subgroup analyses, no differences among studies were observed comparing different types of intervention (*p* = 0.16), types of provider (*p* = 0.40) or control group condition (*p* = 0.45). However, there was a significant difference in the duration of interventions (*p* = 0.001). The largest ES was found in the interventions lasting from 6 to 12 weeks (−0.40).

### 3.7. Mood

**Figure 5:**
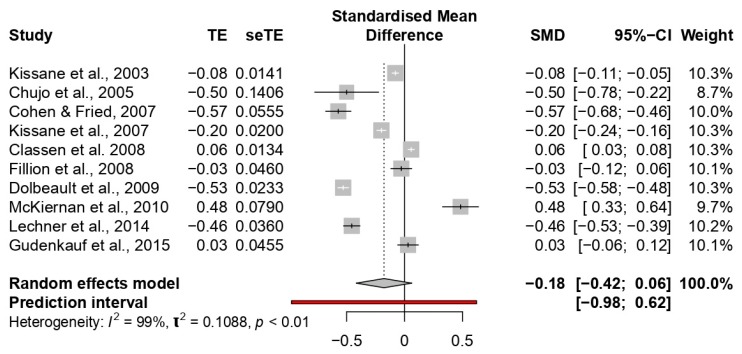
Forest plot of the studies considering psychological interventions focused on increasing mood.

The overall ES in the improvement of mood was small (−0.17; 95% CI: −0.41 to 0.06). The meta-analysis showed high heterogeneity among the studies (*p* < 0.0001; I^2^ = 98%) (see Figure 5).

No study showed a large ES; eight studies reported medium ESs ranging between −0.20 [57] and −0.57 [67]. All the other studies reported small ESs ranging between −0.02 [34] and −0.08 [24].

Subgroup analyses of different treatments on mood did not show significant differences (*p* = 0.41). Moreover, no differences were found when the treatment group (*p* = 0.46) and intervention duration (*p* = 0.11) were considered. However, significant differences were observed when the type of provider (*p* < 0.01) and the control group condition (*p* < 0.0001) were considered. Specifically, higher ESs were observed in the studies with various types of the provider (−0.40; e.g., counsellor, postgraduate student, etc.), and that used standard care in the control group (−0.56) (see Table 3).

### 3.8. Quality of Life

**Figure 6:**
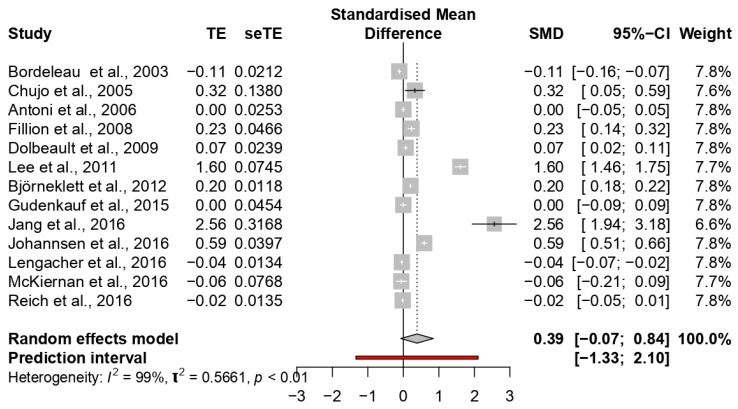
Forest plot of the studies considering psychological interventions focused on increasing quality of life.

The overall ES for improved quality of life was medium (0.38; 95% CI: −0.07 to 0.84). High heterogeneity among the studies (*p* < 0.0001; I^2^ = 98%) was found (see Figure 6).

Two studies showed a large ES, ranging between 1.60 [64] and 2.56 [51]; four studies reported medium ESs ranging between 0.32 [33] and 0.59 [52]; the other studies reported small ESs ranging between 0 [25] and 0.20 [65].

In subgroup analyses, significant differences were observed considering types of treatment (*p* = 0.0001) and types of the provider (*p* = 0.0001). The highest ESs were highlighted in the studies considering CBT interventions (ES = 0.55) and different types of providers (ES = 2.56). No differences emerged considering intervention duration (*p* = 0.30) or control group condition (*p* = 0.15).

## 4. Discussion

The goals of this study were to assess the clinical effectiveness of psychological treatments (psycho-educational intervention, cognitive behavioural therapy or supportive-expressive therapy) on different psychological outcomes (anxiety, depression, mood and quality of life) in women with a breast cancer diagnosis. Overall, the present review showed the low quality of the studies on this topic, characterised by few and heterogeneous methodological information, which influenced the meta-analysis results.

However, our results showed medium effect sizes that indicate moderate effectiveness of the selected approaches in improving the psychological well-being of patients with breast cancer, almost when the psychological variables analysed in this review are considered as the outcomes.

Several subgroup analyses about specific variables (e.g., approach, timing, provider) highlighted an influence of some characteristics of the sample and the methods adopted in the clinical trials, which could have determined the high heterogeneity among the studies as well as the different treatment efficacies.

Considering the effect of psychological interventions on patient anxiety levels, the overall effect size of the treatment (−0.39) was similar to that reported by previous findings, considering the heterogeneous population of patients with cancer (ES = −0.42) [80] or considering specifically breast cancer (ES = 0.40) [35]. Although other studies showed higher effect sizes of the intervention aiming to reduce anxiety in patients with breast cancer [35,36,39,42,80,81], this difference could be related to the choice to analyse only group therapies, disregarding one-to-one interventions [35,36,80,81]. Individual treatments could be more effective in reducing anxiety and improving personal skills aiming to manage the distress related to the cancer condition.

Interestingly, when the outcome was anxiety, it was related to the differences in the efficacy of the approaches considered. The clinical efficacy of cognitive behavioural therapy was found to be comparable to the benefits of psycho-educational interventions. Conversely, supportive-expressive interventions appeared to fail in showing significant clinical benefits in the reduction of anxiety symptoms; these results agree with those observed by previous studies [22,66,82,83]. The effectiveness of cognitive behavioural therapy on anxiety is well known [82], and many studies [22,66,83] highlighted its effects on heterogeneous populations of patients with cancer. Additionally, the psycho-educational approach appears to increase personal medical-condition awareness and its related psycho-physical consequences, such as improved general psychological well-being and decreased anxiety [22,84]. The modification of maladaptive cognitive schemas and the improvement of affective and emotional states are of clinical importance; however, due to the heterogeneity of the studies, also in sub-groups analyses, we cannot generalise our results.

A moderate-to-strong clinical impact in patients with breast cancer appears to be led by different provider types, including various specialist figures (nurse, psychologist and psychiatrist together). We hypothesised that this effect might reflect higher perceived support due to the interaction with more specialists during the treatment.

For the depression outcome, a clinical impact of the treatment (ES = −0.35) was observed. Depression represents an essential, potentially fatal, but treatable complication of breast cancer in women. Depression affects the quality of life of patients and caregivers, and it contributes to lower adherence to medical treatment [85]. Identifying an intervention which allows reducing depressive symptoms related to breast cancer is relevant to improving the quality of life of patients during treatment and in the follow-up phase.

CBT appears to be the best-known treatment for depression [86] because it can help to modify maladaptive information processes associated with negative thinking, which has a causal role in the maintenance of depression. This treatment has superior efficacy to other psychotherapies in the acute treatment of depression and the reduction of depressive symptomatology. Our results also confirmed the effectiveness of CBT in reducing depressive symptoms in patients with breast cancer, proving that the tools furnished by this intervention are more helpful than other treatments (e.g., supportive-expressive group therapy) in counteracting depression [87,88]. These findings highlight the importance of providing CBT to women with breast cancer, and could help to increase adherence to medical treatment.

Considering mood, the meta-analysis showed an overall low effect size (ES = −0.20), although the psycho-educational approach appears to be partially better than the other types of interventions in increasing the mood of the patients (ES = −0.37). Its efficacy could be related to the characteristics of this kind of treatment, focused on the integration of information provision and the promotion of physical activity [40,89], which would seem to favour an increase of mood in stressful situations. In discussing these results, we must consider the assessment tool used in the studies (i.e., the Profile of Mood States; [90]) that analysed different dimensions of the mood (e.g., Stress-Anxiety, Depression-Discomfort, etc.).

Undoubtedly, depression, anxiety and mood—especially if in comorbidity—are bound to impact the quality of life. Therefore, this meta-analysis also investigated the direct impact of therapies on quality of life.

A clinical effect (ES = 0.32) on the quality of life was found, but it was also characterised by a higher heterogeneity. Cognitive behavioural therapy showed stronger effects compared to psycho-educational therapy and supportive-expressive therapy. Both the increase of self-awareness and the development of a non-judgmental attitude can promote better acceptance of the breast cancer diagnosis, and these effects could improve prognosis. Moreover, a more significant therapeutic efficacy in the improvement of the quality of life was observed in treatments made by more than one provider.

Generally, the meta-analysis showed results on the effectiveness of the different approaches on the outcomes considered; however, the high heterogeneity and the low quality of the studies highlighted the low robustness of these results and underlined the importance of developing studies with a better methodological structure. However, the findings of the present study allow us to suggest the development of a treatment including different professional figures, providing a specific role in the therapeutic process to implement the intervention in its different phases. Moreover, a clear definition of the activity of the control groups should also be considered in structuring a well-defined intervention protocol.

The group treatments included in the present study appeared to be more effective than individual ones in the improvement of the psychological outcomes that we have considered [35].

Overall, our findings allow us to conclude that both cognitive behavioural and psycho-educational therapies are associated with significant benefits. These treatments improve the quality of life and the mood, reducing anxiety and depression in women with breast cancer. These results agree with those observed by other studies [35,91] which underlined the effectiveness of group therapy in women with localised breast cancer. These findings could be due to a preference of the patients to enter group therapy, which is characterised by a higher perceived social support.

Another reflection could be made on the choice of some studies to include health education (i.e., information about cancer and treatment and its side-effects) as a sort of “placebo” in the control groups [25,59,62,63]. The positive effect of this type of intervention could have affected the effect size of the analysis. However, the reception of educational materials did not show the same beneficial effect of structured psycho-educational programs, because it is not characterised by a group intervention but by the individual acquisition of notion about the illness condition [92].

This meta-analysis also underlined the confounding effect given by the use of different measures for the assessment of the analysed outcomes. This could have influenced the results of the studies. All the studies used self-reported measures of the outcomes, with the relative limits related to the objectivity of the information. However, there are few direct comparisons of the efficacy of these instruments in the population with breast cancer. Anxiety in people with cancer is commonly assessed through screening questionnaires [93]. Frequently, the analysed studies used the Hospital Anxiety and Depression Scale and the State-Trait Anxiety Inventory (see Table 1). The HADS appears to be more effective than other instruments for the assessment of anxious symptoms in patients with cancer. Conversely, the STAI was used as a screening tool and appeared to be more accurate in the measure of the longitudinal trend of the anxiety [93]. For this reason, the use of the STAI could be recommended for the screening of anxiety. Furthermore, it could be useful to analyse the effectiveness of the intervention in the follow-up. Many self-reported measures are commonly used to assess the depressive symptomatology in patients with breast cancer (see Table 1), although there is a need to identify the elective measure to better define depression in patients with breast cancer. Many investigations on depression in patients with cancer have used the HADS. These studies were mainly focused on identifying the cut-off scores for the diagnosis of depression in patients with cancer [94], even if the HADS represents a self-reported measure explicitly developed for assessing depression in patients within an outpatient medical setting [94]. The use of many instruments for the assessment of the psychological outcomes related to breast cancer and the different dimensions evaluated by the various questionnaires underlines the need to define standardised measures for the analysis of these psychological aspects in this clinical condition. A standardised assessment protocol could help to evaluate the effectiveness of the psychological interventions and could allow better comparison of the efficacy of the different types of psychological therapies.

Treatment duration from six to twelve weeks, compared to briefer or longer interventions, appeared to achieve better results for all the psychological dimensions considered in the present study. This finding is particularly relevant in the clinical setting, and it could also be useful from an economic point of view because a relatively brief psychological treatment could help to reduce psychopathological comorbidity in women with breast cancer and it could improve adherence to medical treatments, with lower direct and indirect costs than long-term treatments.

An interesting result of the present study is related to the good efficacy of cognitive behavioural therapy. Although many studies have shown the effectiveness of this type of intervention in different types of health promotion (for a review see [95]), the higher power of this approach in this meta-analysis could be due to the inclusion of studies used Mindfulness-Based Cognitive Therapy (MBCT). MBCT is an approach that integrates classical cognitive behavioural strategies with relaxation exercises for stress reduction and exercises of body perception. Previous studies found an effect of this therapy in promoting quality of life in patients [96,97,98] and survivors [53,99] of cancer. Further studies are necessary to analyse the differences in the efficacy between standard cognitive behavioural therapies and mindfulness-based cognitive therapy. Moreover, our study considered only a few specific psychological approaches, but further studies could consider the benefits of other types of therapies [88]. For example, it has been shown that hypnosis-based psychotherapy is beneficial to reduce fatigue and cancer-related pain and to control sleep diseases [100,101,102]. Moreover, psychodynamic psychotherapy has resulted in a short-term improvement in patients’ depressive symptoms [103], and interventions based on physical activity seem to improve health in cancer survivors [104]. For these reasons, further studies should consider other therapeutic approaches to define their effectiveness.

### Limitations

The current review and meta-analysis has several limitations. The high heterogeneity of the studies represents the main weakness. The efficacy of the treatments and the comparison of the different approaches showed high variability among the studies. This heterogeneity influenced the power of the studies and the effect size concerning the efficacy of psychological treatments. The heterogeneity of studies, which represents the most impactful limitation of this work, makes generalisation of the results difficult. Most of the studies included a sample with patients at different cancer stages (e.g., stage I, metastatic stage), or with different medical interventions (e.g., chemotherapy, radiotherapy)—all aspects that could influence the psychological outcomes, modulating the effectiveness of the treatment itself. This could suggest a methodological reflection about the lack of a standard protocol in psychological treatments aimed at patients with breast cancer. It could guarantee higher transparency and replicability of the results, according to the results of the qualitative assessment, which showed some concerns about the risk of bias in the studies. This heterogeneity could be due to the choice to not have strictly defined inclusion criteria. This decision was influenced by the fact that this is the first meta-analysis pointed to compare the most common psychological interventions carried out in women with breast cancer. Second, it should be noted that grey literature (i.e., the unpublished studies showing different results) was excluded from this review, and this choice could represent a bias. Although the examination of the follow-up was not one of the objectives of our study, the systematic analysis showed high variability in the timing of follow-up assessments of the interventions. For further research, it could be interesting to analyse the long-term efficacy of the treatments, with a stricter definition of inclusion criteria and studies focused on follow-ups. Another possible selection bias was due to the exclusive consideration of group therapies without a comparison with individual ones.

Finally, the medical treatment of breast cancer has significantly changed in recent years, with the advent of new systemic treatments [105]. According to these therapeutic changes, psychological therapies have also changed. 

This review suggests that psycho-educational, supportive-expressive and cognitive behavioural therapies are the gold-standard treatments for women with breast cancer. This conclusion is supported by their roles in the treatment of perseverative thinking, attentional biases and metacognitive processes [105,106] that are involved in determining the outcomes analysed in the present systematic review and meta-analysis.

## 5. Conclusions

This review and meta-analysis confirms the efficacy of cognitive behaviour and psycho-educational therapies in both the modification of maladaptive cognitive schemas and the improvement of affective and emotional states. These treatments are of clinical importance to reduce anxiety and depression and to improve mood and quality of life in women with breast cancer.

Further studies should control methodological features and should structure a protocol able to enhance internal validity and provide more reliable findings.

Despite these limits, this review and meta-analysis could guide the development of new psychological interventions or help to optimize the efficacy of existing ones aimed at improving perceived well-being and quality of life. 

## Figures and Tables

**Table 1:** Information about the studies included in the meta-analysis. For each study, the following variables are reported: the outcome and the instruments (Tools) utilised to evaluate it; the sample size (*n*); the Intervention Type; the Intervention Duration; the Control Condition; the Inclusion Criteria; the Provider; and the Stage of Cancer.

Variables	Tools	Study	*n* (Treatment/Control)	Intervention Type	Intervention Duration	Control Condition	Inclusion Criteria	Provider	Stage of Cancer
*Anxiety*	HADS	Kissane et al. [24]	145/141	CBT + Relaxation Lessons	20 weeks	Relaxation Lessons	Inpatient Treatment	Two Therapists	I; II
	STAI	Dolbeault et al. [32]	85/93	PET	8 weeks	Wait List	After Treatment	Two Therapists	I; II; III
	HADS	Groarke et al. [50]	102/120	CB-CBSM	5 weeks	Usual Care	After Treatment	Clinical Psychologist	From 0 to IV
	HADS	Ho et al. [30]	47/51	SET	8 weeks	Social Support	After Treatment	Therapist	I; II; III
	PAI	Jang et al. [51]	12/11	CBT-MBSR	12 weeks	Wait List	After Treatment	Mental Health Specialist	From 0 to III
	HADS	Johannsen et al. [52]	46/61	CBT-MBSR	8 weeks	Wait List	After Treatment	Mindfulness Instructor	Not Reported
	STAI	Lengacher et al. [53]	152/147	CBT-MBSR	6 weeks	Usual Care	After Treatment	Clinical Psychologist	From 0 to III
	STAI	Reich et al. [54]	159/152	CBT-MBSR	6 weeks	Usual Care	After Treatment	Clinical Psychologist	From 0 to III
	STAI	Zhang et al. [55]	28/30	CBT-MBSR	8 weeks	Usual Care	After Treatment	Psychologist, Expert in Mindfulness	I; II; III
	HADS	Kenne Sarenmalm et al. [56]	62/52	CBT-MBSR	8 weeks	Non-MBSR	After Treatment	Mindfulness Instructor	Not Reported
*Depression*	HADS	Kissane et al. [24]	145/141	CBT + Relaxation Lessons	20 weeks	Relaxation Lessons	Inpatient Treatment	Two Therapists	I; II
	BDI	Kissane et al. [57]	115/56	SET	13 weeks	Relaxation Lessons	After Treatment	Therapist	IV
	HADS	Groarke et al. [50]	102/120	CBT-CBSM	5 weeks	Usual Care	After Treatment	Clinical Psychologist	From 0 to IV
	CES-D	Bower et al. [58]	32/33	CBT-MAP	6 weeks	Wait List	After Treatment	Not Reported	From 0 to III
	FACT	Lechner et al. [59]	57/57	CBT-CBSM	10 weeks	Educational Program	After Treatment	Clinical Psychologist	From 0 to IV
	CES-D	Dodds et al. [60]	16/12	CBT-CDCT	16 weeks	Wait List	After Treatment	Clinically Trained PhD and Social Work Researcher	Not Reported
	HADS	Ho et al. [30]	47/51	SET	8 weeks	Social Support	After Treatment	Therapist	I; II; III
	PAI	Jang et al. [51]	12/11	CBT-MBSR	12 weeks	Wait List	After Treatment	Mental Health Specialist	From 0 to III
	CES-D	Lengacher et al. [53]	152/147	CBT-MBSR	6 weeks	Usual Care	After Treatment	Clinical Psychologist	From 0 to III
	CES-D	Reich et al. [54]	154/146	CBT-MBSR	6 weeks	Usual Care	After Treatment	Clinical Psychologist	From 0 to III
	CES-D	Boyle et al. [61]	35/30	CBT-MAP	6 weeks	Wait List	After Treatment	Not Reported	From 0 to III
	HADS	Kenne Sarenmalm et al. [56]	62/52	CBT-MBSR	8 weeks	Non-MBSR	After Treatment	Mindfulness Instructor	Not Reported
*Quality of Life*	EORTC-C30	Bordeleau et al. [62]	145/70	SET + Educational Training	12 weeks	Educational and Relaxation Training	After Treatment	Psychiatrist, Psychologist, Social Workers, Nurse	IV
	EORTC-C30	Chujo et al. [33]	22/11	PET	6 weeks	Wait List	After Treatment	Psychiatrist and Nurse	Not Reported
	ABS	Antoni et al. [25]	74/85	CBT	10 weeks	Educational Program	After Treatment	Psychologist	From 0 to III
	SF-12	Fillion et al. [34]	44/43	PET	4 weeks	General Information	After Treatment	Nurses	From 0 to III
	EORTC-C30	Dolbeault et al. [32]	81/87	PET	8 weeks	Wait List	After Treatment	Two Therapists	I; II; III
	EORTC-C30	McKiernan et al. [63]	23/30	CBT + Educational Training	6 weeks	Educational Training	After Treatment	Clinical Psychologist and Counselling Psychologist	I; II; Metastasis and Lymph
	QoL Scales for Korean Cancer Patients	Lee et al. [64]	35/36	CBT	6 weeks	Not Reported	After Treatment	Nurse	I; II; III
	EORTC-C30	Bjorneklett et al. [65]	176/164	PET	1 week + 4 meetings after 2 months	Wait List	After Treatment	Not Reported	I; II; III
	FACT	Gudenkauf et al. [66]	40/49	CBT + Relaxation Training	5 weeks	Relaxation Training	After Treatment	Clinical Psychology student	From 0 to III
	EORTC-C30	Jang et al. [51]	12/11	CBT-MBSR	12 weeks	Wait List	After Treatment	Mental Health Specialist	From 0 to III
	WHO-5	Johannsen et al. [52]	46/61	CBT-MBSR	8 weeks	Wait List	After Treatment	Mindfulness Instructor	Not Reported
	SF-36	Lengacher et al. [53]	152/147	CBT-MBSR	6 weeks	Usual Care	After Treatment	Clinical Psychologist	From 0 to III
	SF-36	Reich et al. [54]	152/145	CBT-MBSR	6 weeks	Usual Care	After Treatment	Clinical Psychologist	From 0 to III
*Mood*	ABS	Kissane et al. [24]	145/141	CBT + Relaxation Training	20 weeks	Relaxation Training	Inpatient Treatment	Two Therapists	I; II
	POMS	Chujo et al. [33]	22/11	PET	6 weeks	Wait List	After Treatment	Psychiatrist and Nurse	Not Reported
	BSI	Cohen and Fried [67]	38/37	CBT + Relaxation Training	9 weeks	Usual Care	After Treatment	Cohen (Expert in Psycho-Oncology)	I; II
	IES	Kissane et al. [57]	115/56	SET	13 weeks	Relaxation Training	After Treatment	Therapist	IV
	POMS	Classen et al. [27]	151/148	SET	12 weeks	Educational Program	After Treatment	Nurse, Psychologist, Social Workers	From 0 to III
	POMS	Fillion et al. [34]	44/43	PET	4 weeks	General Information	After Treatment	Nurses	From 0 to III
	POMS	Dolbeault et al. [32]	81/87	PET	8 weeks	Wait List	After Treatment	Two Therapists	I; II; III
	POMS	McKiernan et al. [63]	23/30	CBT + Educational Training	6 weeks	Educational Training	After Treatment	Clinical Psychologist and Counselling Psychologist	I; II; Metastasis and Lymph
	POMS	Lechner et al. [59]	57/57	CBT-CBSM	10 weeks	Educational Program	After Treatment	Clinical Psychologist	From 0 to IV
	ABS	Gudenkauf et al. [66]	40/49	CBT + Relaxation Training	5 weeks	Relaxation Training	After Treatment	Clinical Psychology student	From 0 to III

ABS: Affect Balance Scale; BDI: Beck Depression Inventory; BSI: Brief Symptom Inventory; CBT-CBSM: Cognitive Behavioural Therapy—Cognitive Behavioural Stress Management; CBT-MBSR: Cognitive Behavioural Therapy—Mindfulness-Based Stress Reduction Program; CBT-MAP: Cognitive Behavioural Therapy—Mindful Awareness Practices; CBT-CDCT: Cognitively-Based Compassion Training; CES-D: Centre for Epidemiologic Studies Depression Scale; EORTC-C30: European Organisation for Research and the Treatment of Cancer Core Quality of Life Questionnaire; FACT: Functional Assessment of Cancer Therapy; HADS: Hospital Anxiety and Depression Scale; MCS-12: Medical Outcomes Studies Short-Form General Health Survey; PAI: Personality Assessment Inventory; SF-12/SF-36: Short-Form Health Survey; PET: psycho-educational therapy; POMS: Profile of Mood States; SET: supportive-expressive group therapy; STAI: State-Trait Anxiety Inventory; WHO-5: World Health Organisation-5 Well-Being Index; IES: Impact of Events Scale.

**Table 2:** Risk of bias assessment for each study selected for the meta-analysis.

Study	Intervention Type	Random Sequence Generation	Allocation Concealment	Blinding (Outcome Assessment)	Incomplete Outcome	Selective Reporting	Other Sources of Bias	Overall Risk of Bias
Bordeleau et al. [62]	SET	+	?	?	?	+	?	?
Kissane et al. [24]	CBT	+	+	+	?	?	?	?
Chujo et al. [33]	PET	−	−	?	−	+	?	−
Antoni et al. [23]	CBT	?	?	+	+	?	?	?
Cohen and Fried [67]	CBT	?	?	+	+	+	?	?
Kissane et al. [57]	SET	+	?	−	?	?	?	−
Classen et al. [27]	SET	+	?	?	?	−	?	−
Fillion et al. [34]	PET	?	?	?	+	+	?	?
Dolbeault et al. [32]	PET	+	+	?	?	+	?	?
McKiernan et al. [63]	CBT	−	−	?	?	?	?	−
Lee et al. [64]	CBT	−	−	−	?	?	?	−
Björneklett et al. [65]	PET	+	+	+	?	+	?	?
Groarke et al. [50]	CBT	+	+	?	+	+	?	?
Bower et al. [58]	CBT	?	?	?	+	+	?	?
Lechner et al. [59]	CBT	?	?	?	+	+	?	?
Dodds et al. [60]	CBT	+	?	?	?	+	?	?
Gudenkauf et al. [66]	CBT	+	+	+	?	+	?	?
Ho et al. [30]	SET	+	?	?	+	+	?	?
Jang et al. [51]	CBT	?	?	?	+	+	?	?
Johannsen et al. [52]	CBT	+	?	?	+	+	?	?
Langacher et al. [53]	CBT	+	?	?	+	+	?	?
Reich et al. [54]	CBT	?	?	−	+	?	?	−
Zhang et al. [55]	CBT	?	−	?	+	+	?	−
Boyle et al. [61]	CBT	−	?	?	+	?	?	−
Kenne Sarenmalm et al. [56]	CBT	+	+	?	+	+	?	?

(+) = low risk of bias; (−) = high risk of bias; (?) = unclear risk of bias.

**Table 3:** Effect size (Cohen’s d) for each variable.

Analyses	Number of Studies	Effect Size (95% CI)	Heterogeneity (I^2^%)
**Anxiety**			
Overall Effect Size	10	−0.39 [−0.91, 0.14]	98
Type of Intervention			-
Cognitive Behavioural Therapy	8	−0.45 [−1.14, 0.24]	97
Psycho-Educational Therapy	1	−0.45 [−0.49, −0.40]	-
Supportive-Expressive Therapy	1	0.10 [0.02, 0.18]	
Type of Provider			
Psychologist	6	−0.14 [−0.31, 0.02]	95
Psychologist + Psychiatrist	1	−0.45 [−0.49, −0.40]	-
Nurse	-	-	-
Other	3	−0.94 [−4.47, 2.58]	98
Not Reported			
Duration of Intervention			
Less than 6 Weeks	1	−0.19 [−0.22, −0.15]	-
Between 6 and 12 Weeks	8	−0.45 [−1.15, 0.26]	98
More than 12 Weeks	-	-	-
Not Reported	1	−0.20 [−0.23, −0.18]	-
Control Group			
Alternative Intervention	3	0.003 [−0.46, 0.47]	98
Wait List	3	−1.12 [−4.16, 1.90]	93
Standard Care	4	−0.18 [−0.40, 0.04]	93
Not Reported			
Inclusion Criteria			
In Patient	1	−0.21 [−0.24, −0.18]	
After Treatment	9	−0.41 [−1.01, 0.19]	98
Not Reported	-	-	-
**Depression**			
Overall Effect Size	12	−0.35 [−0.79, −0.10]	98
Type of Intervention			
Cognitive Behavioural Therapy	10	−0.42 [−0.96, 0.12]	98
Psycho-Educational Therapy	-	-	-
Supportive-Expressive Therapy	2	−0.10 [−2.18, 2.16]	98
Type of Provider			
Psychologist	7	−0.15 [−0.37, 0.06]	97
Psychologist + Psychiatrist	-	-	-
Nurse	-	-	-
Other	3	−1.04 [−3.85, 1.76]	97
Not Reported	2	−0.08 [−9.44, 9.26]	99
Duration of Intervention			
Less than 6 Weeks	1	−0.08 [−0.11, −0.04]	-
Between 6 and 12 Weeks	8	−0.40 [−1.12, 0.33]	98
More than 12 Weeks	1	−0.18 [−0.21, −0.14]	-
Not Reported	2	−0.39 [−4.31, 3.51]	93
Control Group			
Alternative Intervention	5	−0.18 [−0.53, 0.17]	98
Wait List	4	−0.79 [−2.72, 1.14]	99
Standard Care	3	−0.11 [−0.16, −0.05]	50
Not Reported	-	-	-
Inclusion Criteria			
In Patient	1	−0.10 [−0.13, −0.08]	
After Treatment	11	−0.37 [−0.86, 0.12]	98
Not Reported	-	-	-
**Mood**			
Overall Effect Size	10	−0.17 [−0.41, 0.06]	98
Type of Intervention			
Cognitive Behavioural Therapy	5	−0.12 [−0.64, 0.39]	98
Psycho-Educational Therapy	3	−0.34 [−1.05, 0.36]	97
Supportive-Expressive Therapy	2	−0.07 [−1.70, 1.56]	99
Type of Provider			
Psychologist	6	−0.12 [−0.51, 0.25]	99
Psychologist + Psychiatrist	-	-	-
Nurse	1	−0.02 [−0.11, 0.06]	-
Other	3	−0.33 [−1.19, 0.54]	98
Not Reported			
Duration of Intervention			
Less than 6 weeks	2	0.001 [−0.36, 0.37]	0
Between 6 and 12 Weeks	5	−0.31 [−0.87, 0.24]	96
More than 12 Weeks	2	−0.07 [−1.70, 1.56]	99
Not Reported	1	.0.08 [−0.10, −0.05]	99
Control Group			
Alternative Intervention	6	−0.03 [−0.35, 0.29]	98
Wait List	3	−0.34 [−1.06, 0.36]	96
Standard Care	1	−0.57 [−0.68, −0.46]	-
Not Reported			
Inclusion Criteria			
In Patient	1	−0.08 [−0.10, 0.05]	-
After Treatment	9	−0.18 [−0.46, 0.08]	98
Not Reported	-	-	-
**Quality of Life**			
Overall Effect Size	13	0.38 [−0.07, 0.84]	98
Type of Intervention			
Cognitive Behavioural Therapy	8	0.55 [−0.25, 1.35]	99
Psycho-Educational Therapy	4	0.17 [0.02, 0.32]	89
Supportive-Expressive Therapy	1	−0.11 [−0.15, −0.07]	-
Type of Provider			
Psychologist	6	0.08 [−0.18, 0.34]	93
Psychologist + Psychiatrist	2	−0.03 [−1.17, 1.12]	96
Nurse	3	0.72 [−1.18, 2.63]	99
Other	1	2.56 [1.93, 3.18]	-
Not Reported	1	0.20 [0.17, 0.22]	-
Duration of Intervention			
Less than 6 Weeks	2	0.11 [−1.34, 1.57]	97
Between 6 and 12 Weeks	10	0.46 [−0.15, 1.08]	90
More than 12 Weeks	-	-	-
Not Reported	1	0.20 [0.17, 0.22]	-
Control Group			
Alternative Intervention	6	0.27 [−0.41, 0.96]	98
Wait List	5	0.71 [−0.53, 1.96]	97
Standard Care	2	−0.03 [−0.18, 0.12]	36
Not Reported	-	-	-
Inclusion Criteria			
In Patient	-	-	-
After treatment	13	0.38 [−0.07, 0.84]	98
Not Reported	-	-	-
